# Pharmacological Insights and Technological Innovations in *Curcuma longa* L. and *Echinacea purpurea* (L.) Moench as Plant-Derived Immunomodulators

**DOI:** 10.3390/ph19010093

**Published:** 2026-01-03

**Authors:** Juan Pablo Espinoza, Valentina Guajardo, Maité Rodríguez-Díaz, Mabel Moreno, Carolina Klagges, Mario Castillo-Ruiz, María Carolina Otero

**Affiliations:** 1 Centro Integrativo de Biología y Química Aplicada (CIBQA), Universidad Bernardo O’Higgins, Santiago 8370854, Chile; jespinoza@ubo.cl; 2 Escuela de Química y Farmacia, Facultad de Medicina, Universidad Andres Bello, Santiago 8370146, Chile; v.guajardoatala@uandresbello.edu; 3 Departamento de Química, Facultad de Ciencias Naturales, Matemática y del Medio Ambiente, Universidad Tecnológica Metropolitana, Las Palmeras 3360, Santiago 7800003, Chile; m.rodriguezd@utem.cl; 4 Instituto de Investigación Interdisciplinar en Ciencias Biomédicas SEK, Facultad de Ciencias de la Salud, Universidad SEK, Santiago 7520317, Chile; mabel.moreno@zonavirtual.uisek.cl (M.M.); carolina.klagges@zonavirtual.uisek.cl (C.K.); 5 Fachbereich Ingenieur-und Naturwissenschaften, Hochschule Merseburg—University of Applied Sciences, Eberhard-Leibnitz-Str. 2, 06217 Merseburg, Germany; 6 Escuela de Tecnología Médica, Departamento de Ciencias Químicas y Biológicas, Facultad de Ciencias de la Salud, Universidad Bernardo O’Higgins, Santiago 8370854, Chile

**Keywords:** immunomodulators, nanotechnology, *Curcuma longa* L., *Echinacea purpurea* (L.) Moench, bioavailability

## Abstract

Immune dysregulation and chronic inflammation are central contributors to many diseases. *Curcuma longa* L. and *Echinacea purpurea* (L.) Moench are widely used medicinal plants with extensive preclinical evidence supporting immunomodulatory effects. Their key metabolites, curcuminoids, turmerones, alkamides, polysaccharides, and caffeic acid derivatives, engage with critical pathways, including NF-κB, MAPK, JAK/STAT, and Nrf2. This interaction modulates cytokine production, oxidative stress responses, and both innate and adaptive immune activities. Although numerous mechanistic and early clinical studies support these actions, human evidence remains inconsistent, partly due to poor and variable oral bioavailability and substantial heterogeneity in extract composition, despite the existence of some standardized preparations. Recent technological strategies, including micelles, phytosomes, phospholipid complexes, nanoemulsions, polymeric nanoparticles, and liposomal systems, have improved solubility, stability, and systemic exposure of key metabolites, particularly curcuminoids. However, clinical results are still limited and often derived from small or heterogeneous trials. This review summarizes the ethnopharmacological background, mechanistic data, clinical findings, and formulation advances for both species and highlights the translational barriers that restrict their therapeutic application. Rigorous clinical studies using standardized and technologically optimized preparations are required to determine the true immunomodulatory potential of *C. longa* and *E. purpurea.*

## 1. Introduction

The immune system has become an increasingly important therapeutic target in recent years, as global health challenges have underscored the importance of maintaining immune balance rather than merely enhancing immune activation [1]. Within this context, the use of plant-derived bioactive compounds as immunomodulators has a long and respected tradition, supported by both ethnopharmacological practices and modern biomedical research [2]. Among these, *Curcuma longa* L. (turmeric) and *Echinacea purpurea* (L.) *Moench* (purple coneflower) stand out as two of the most extensively studied medicinal species for their ability to regulate immune responses, attenuate inflammation, and protect against infectious and chronic diseases [3]. However, a wide range of other medicinal plants, including *Andrographis paniculata*, *Astragalus* spp., *Nigella sativa*, *Zingiber officinale*, *Glycyrrhiza glabra*, and *Lentinula edodes*, have also demonstrated significant immunomodulatory and anti-inflammatory activities and are extensively used in traditional medicine and contemporary dietary supplementation [4]. The present review focuses on *C. longa* and *E. purpurea* due to the exceptional breadth of mechanistic, formulation-based, and clinical evidence available for these two species, rather than implying exclusivity in their biological relevance. Their traditional use in Asian and Western herbal medicine has evolved into a subject of intense scientific scrutiny, with an expanding number of preclinical and clinical studies supporting their immunomodulatory potential.

Although synthetic immunomodulators such as corticosteroids and biologics remain essential in clinical practice, they have limitations such as systemic toxicity, high cost, and uncertain long-term safety [5]. These drawbacks have intensified the search for natural alternatives that can modulate the immune system more safely [6].

Despite increasing scientific evidence supporting the benefits of curcuminoids from *C. longa* and of caffeic acid derivatives and alkylamides from *E. purpurea*, several challenges hinder their clinical translation. These compounds have limitations such as poor aqueous solubility, chemical instability, low intestinal absorption, rapid metabolism, and variability in phytochemical composition between batches [7,8]. These limitations largely account for the disparity between the promising results observed in preclinical studies and the inconsistent outcomes seen in clinical trials.

In response, technological innovations have emerged as key drivers in improving the pharmacological performance of these plant compounds [9]. Advances in pharmaceutical and nanotechnological systems, including nanoparticles, liposomes, nanoemulsions, and solid lipid carriers, are being complemented by encapsulation in biopolymers or cyclodextrins and by synergistic co-formulations with bioenhancers such as piperine to enhance bioavailability, stability, and targeted delivery [10]. At the same time, biotechnological approaches such as plant cell culture engineering, metabolic pathway optimization, and the use of solvent-free “green extraction” methods have opened new possibilities for the standardized and sustainable production of active compounds on a large scale. However, the development of high-bioavailability formulations has also raised new safety concerns. Case series from Drug-Induced Liver Injury (DILI) registries and individual reports now describe acute drug-induced liver injury associated with turmeric or curcumin supplements, particularly products co-formulated with the bioenhancer piperine or other agents that markedly increase systemic exposure. In several cases, latency of a few weeks to months, exclusion of alternative causes, and clinical improvement after withdrawal support a causal role for these supplements. Regulatory authorities in some countries have issued warnings or updated product labeling in response to rising reports of turmeric-related hepatotoxicity. Although nano-curcumin and lipid-based carriers can improve pharmacokinetics and show hepatoprotective effects in experimental models, they may also shift tissue exposure far beyond that achieved with traditional preparations, underscoring the need for rigorous, formulation-specific safety evaluation rather than assuming a class effect [11,12].

This narrative review is based on literature retrieved primarily from PubMed, Scopus, and Web of Science, including in vitro, in vivo, and clinical studies, as well as systematic reviews evaluating extracts or derived products of *C. longa* and *E. purpurea*. The objective of this review is to critically integrate phytochemical, mechanistic, clinical, and formulation-based evidence for both species and to identify the key translational barriers that limit their therapeutic application. By combining ethnopharmacological knowledge with contemporary insights into molecular mechanisms and formulation science, this review provides an updated and evidence-based framework for understanding the immunomodulatory potential of *C. longa* and *E. purpurea*, while outlining the advances and limitations that shape their future clinical relevance [13].

## 2. Materials and Methods

A comprehensive literature search was performed using the NCBI–PubMed, Google Scholar, and Mendeley databases. Search terms included “immunomodulators,” “nanotechnology,” “*Curcuma longa* L.,” “*Echinacea purpurea* (L.) Moench,” “bioavailability,” “medical applications,” and “nanomedicine,” individually and in combination using Boolean operators. Specific applications (e.g., “nanotechnology+ *Curcuma longa* L.,” “immunomodulators + *Echinacea purpurea* (L.) Moench” and “*Echinacea purpurea* (L.) Moench + citoxicity”) were also explored to ensure thematic coverage.

The inclusion window spanned 1989–2025, capturing both early foundational discoveries and recent advances. Only peer-reviewed journal articles, including original research, reviews, and meta-analyses, were considered. The exclusion criteria involved conference abstracts, non-peer-reviewed content, and papers not directly related to *C. longa* or *E. purpurea*.

After a thorough screening and manual curation for relevance, methodological rigor, and application specificity, a total of 151 scientific references were selected. These references were carefully analyzed concerning plant composition, biological properties, immune system applications, biodistribution, biocompatibility, and therapeutic efficacy. This synthesis offers a comprehensive overview of *C. longa* and *E. purpurea* as promising phytopharmaceuticals in the field of translational medicine.

Inclusion Criteria:

Preclinical and clinical studies investigating curcumin and echinacea were included if they focused on participants with autoimmune or inflammatory diseases, such as rheumatoid arthritis and multiple sclerosis, as well as healthy individuals undergoing immunomodulatory assessments. Eligible studies were required to evaluate primary interventions involving curcumin or echinacea and their derivatives, utilizing effective dosages and measuring specific immunomodulatory outcomes, such as changes in inflammatory markers and immune cell activity. Additionally, included studies employed rigorous methodologies such as randomized controlled trials, double-blind designs, or systematic reviews, and were limited to peer-reviewed publications from 2010 to 2025.

Exclusion Criteria:

Studies were excluded if they involved participants with known contraindications to either curcumin or echinacea, or those taking medications that could potentially interact. Furthermore, studies utilizing inadequate dosages or lacking a robust design were not considered. Research that failed to adequately assess relevant immunological effects or did not present clear, isolated data on curcumin or echinacea was also excluded from this review.

## 3. Immunomodulation as a Therapeutic Strategy

The immune system plays a central role in maintaining physiological homeostasis by orchestrating defense mechanisms against pathogens while preventing excessive inflammatory damage. Dysregulation of immune responses, whether through hyperactivation or suppression, has been linked to a wide range of acute and chronic conditions, including autoimmune diseases, metabolic disorders, infections, and cancer [14]. Consequently, the concept of immunomodulation has emerged as a critical therapeutic approach aimed at restoring immune balance rather than merely stimulating or inhibiting immune responses.

Immunomodulation refers to the modulation of the immune response through biological or chemical agents that can enhance, suppress, or normalize immune functions depending on the pathological context [5]. Conventional synthetic immunomodulators, such as corticosteroids, calcineurin inhibitors, and biologic agents targeting cytokines (e.g., TNF-α inhibitors), are effective but often associated with significant drawbacks, including systemic toxicity, high production costs, and limited long-term tolerability [5,15,16]. Prolonged use of these treatments may lead to immunosuppression, opportunistic infections, and metabolic side effects, underscoring the urgent need for safer and more integrative alternatives.

In this context, natural immunomodulators derived from plants have attracted growing attention due to their multi-target mechanisms and favorable safety profiles. Phytochemicals, including polyphenols, terpenoids, alkaloids, and polysaccharides, are known to modulate key signaling pathways such as NF-κB, MAPK, and Nrf2, which are involved in inflammation, oxidative stress, and immune regulation [2]. Unlike single-target synthetic drugs, plant-derived compounds exhibit pleiotropic effects, influencing both innate and adaptive immunity. This dual modulation allows them to fine-tune macrophage activation (M1/M2), regulate T-helper polarization (Th1/Th2/Th17/Treg) and adjust cytokine production toward a balanced immune state [17].

Furthermore, the post-pandemic scenario has highlighted the importance of immunomodulatory strategies in preventing and mitigating viral and inflammatory diseases. Natural products capable of enhancing antiviral defenses, while controlling excessive inflammation have been explored as supportive interventions in respiratory and systemic infections [1]. In this context, *Curcuma longa* L. and *Echinacea purpurea* (L.) Moench have emerged as exemplary species. Their bioactive metabolites, including curcuminoids, alkylamides, and caffeic acid derivatives, are known to modulate innate immune cells such as macrophages and dendritic cells, as well as adaptive responses mediated by T and B lymphocytes [18,19]. These actions collectively contribute to a balanced immune response characterized by a reduction in pro-inflammatory cytokines (e.g., IL-6, TNF-α) and by enhanced immune surveillance [18,19].

The concept of immunomodulation aligns with the emerging paradigm of network pharmacology, which recognizes that complex diseases require agents capable of modulating multiple molecular targets simultaneously. This has sparked renewed interest in standardized phytotherapeutics and nutraceuticals as complementary strategies for immune-related disorders. However, despite promising preclinical and clinical findings, the translation of plant-based immunomodulators into mainstream medicine is still limited by pharmacokinetic constraints and variability in extract composition, challenges that emerging formulation technologies seek to address [5,20].

Overall, immunomodulation represents a multifaceted therapeutic strategy aimed at restoring immune equilibrium by modulating specific molecular pathways. Plant-derived bioactives, such as those from *C. longa* and *E. purpurea*, illustrate the potential of natural compounds to combine pharmacological efficacy with improved safety and sustainability. Research on these species integrates traditional medical knowledge with advances in molecular immunology and formulation science, highlighting their promise as models for developing safer and more effective plant-based immunotherapeutic approaches.

### Immunomodulation of C. longa and E. purpurea

In *C. longa*, polyphenolic curcuminoids (two aromatic rings connected by a conjugated enone linker) and sesquiterpene ketones (turmerones), modulate immunity by inhibiting NF-κB and pro-inflammatory mediators, activating antioxidant defenses via Nrf2 pathways, and regulating macrophage and dendritic cell functions [21]. In *E. purpurea*, caffeic acid derivatives (e.g., echinacoside), classified as polyphenolic glycosides, together with lipophilic alkylamides, enhance immune responses by stimulating phagocytic activity, enhancing NK-cell cytotoxicity, and modulating cytokine production, including interleukins and interferons [22]. The polysaccharides of *E. purpurea*, such as arabinoxylan glucans (PS I and PS II), are key to its immunomodulatory action, activating phagocytosis, improving leukocyte mobility, and stimulating cytokine production, thus strengthening innate and adaptive immunity, although they work in synergy with alkylamides and phenolic acids for their immunostimulatory and anti-inflammatory effect. Overall, metabolites from both species support innate and adaptive immunity, highlighting their therapeutic potential for the management of inflammation and immune dysregulation [23] (Table 1).

In various experimental models, turmeric preparations often reduce pro-inflammatory mediators while supporting regulatory responses, consistent with the inhibition of NF-κB and its downstream effectors, the activation of Nrf2-dependent antioxidant pathways, and the modulation of macrophage and dendritic cell activities [24]. Echinacea extracts have been shown to enhance immune function by stimulating phagocytic activity and increasing NK-cell cytotoxicity. Additionally, these extracts can modulate cytokine production, including upregulation of key interleukins and interferons, thereby contributing to finely tuned immune responses that promote anti-inflammatory effects while enhancing pathogen resistance. This immunomodulatory action is mediated through multiple cellular pathways, thereby enhancing both innate and adaptive immunity [22,25,26]. Clinical findings remain heterogeneous yet generally align with these mechanistic observations, suggesting potential benefits in conditions characterized by inflammation and dysregulated host defense. At the same time, variability in phytochemical content, limited standardization, and low oral bioavailability limit the reproducibility of outcomes [22,25,26]. These constraints motivate the technological strategies discussed in subsequent sections of this review, where formulation science and bioengineering are employed to enhance stability, absorption, and targeted delivery.

**Table 1:** Main bioactive metabolites of *C. longa* and *E. purpurea* and their immunomodulatory effects.

Phytochemicals Class	Main Metabolites/Chemical Structure	Immunomodulatory Mechanism	Plant Species	References
Polysaccharide	These are complex and diverse molecules, not uniform, with different types of sugars and degrees of branching. PS I(an arabino-xylan) and PS II (containing rhamno-arabino-galactan)	They activate phagocytosis, improve leukocyte motility, and stimulate cytokine production, thus strengthening innate and adaptive immunity.	*E. purpurea*	[26,27]
Phenylpropanoid	Echinacoside	Stimulation of phagocytic activity and cytokine production (ILs, IFNs); enhancement of antioxidant response.	*E. purpurea*	[26,27]
	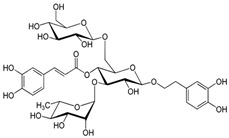			
Phenylpropanoid	Chicoric acid	Stimulation of phagocytic activity and cytokine production	*E. purpurea*	[26,27]
	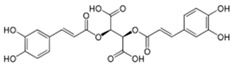			
Triterpenic saponins	Sugar attached to triterpene or steroid aglycone. Ej: Oleanane-type saponins	Enhancement of macrophage and NK cell activity; promotion of adaptive immune responses.	*E. purpurea*	[26,27]
	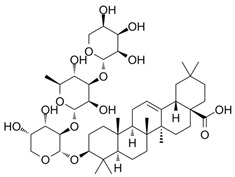			
Lipophilic amides	Alkylamides	Interaction with cannabinoid receptors (CB2); regulation of cytokine synthesis; modulation of innate immune response.	*E. purpurea*	[22,27]
	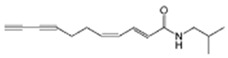			
Polyphenols	Aromatic aliphatic ring containing hydroxyl groups Ej: curcumin	Inhibition of NF-κB and pro-inflammatory mediators; activation of antioxidant defenses via Nrf2; regulation of macrophage and dendritic cell activity.	*C. longa*	[22,23]
	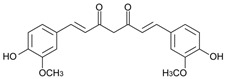			
Terpenes (volatile oils)	Turmerones (α-, β-, ar-, Ej: ar-turmerone)	Modulation of immune cell signaling and cytokine release; suppression of inflammatory responses.	*C. longa*	[22,28]
	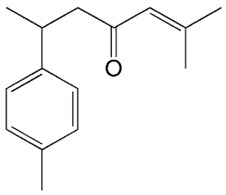			

## 4. *Curcuma longa* L.

### 4.1. Ethnopharmacological Insights

*Curcuma longa* L., commonly known as turmeric, is a perennial herbaceous plant of the family Zingiberaceae. Native to South and Southeast Asia, it is widely cultivated across tropical and subtropical regions, with India being the leading global producer, followed by China, Indonesia, Sri Lanka, Bangladesh, Thailand, and certain regions of Africa and South America. The plant grows up to one meter tall and produces underground rhizomes that serve as the primary reservoir of its bioactive compounds. These rhizomes are rich in curcuminoids, which confer the characteristic yellow pigmentation, as well as in essential oils and other secondary metabolites of pharmacological relevance [29].

The use of turmeric in traditional medicine has been documented for over 4000 years, with substantial evidence from ancient Ayurvedic, Siddha, Unani, and Traditional Chinese Medicine (TCM) systems. In Ayurveda, turmeric was prescribed for digestive disorders, skin conditions, respiratory diseases, and inflammatory ailments. In TCM, where it is known as Jianghuang (姜黄), the traditional Chinese name for turmeric, it has traditionally been used to promote blood circulation, relieve pain, and reduce swelling, particularly in musculoskeletal and joint-related conditions. Beyond medicine, turmeric has been employed as a culinary spice, natural dye, preservative, and as part of religious and cultural practices across various civilizations [30,31,32].

Today, turmeric is not only a vital ingredient in global cuisine but also serves as a valuable raw material for nutraceuticals and phytopharmaceuticals. Its rich ethnopharmacological history, bolstered by contemporary pharmacological research, positions *C. longa* as a key species that bridges traditional practices with modern biomedical investigations [29]. This dual significance provides the foundation for exploring its immunomodulatory properties and leveraging technological advancements to enhance its therapeutic potential.

### 4.2. Pharmacological and Immunomodulatory Profile

**Figure 1:**
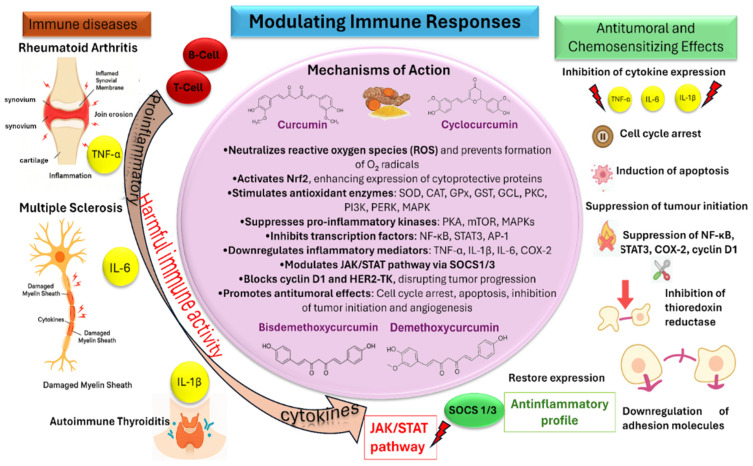
Immunomodulatory and antitumoral mechanisms of curcumin and its derivatives, demethoxycurcumin, bisdemethoxycurcumin, and cyclocurcumin. These compounds modulate immune responses by neutralizing reactive oxygen species (ROS), activating Nrf2 to induce cytoprotective proteins, and stimulating antioxidant enzymes (SOD, CAT, GPx, GST, GCL, PKC, PI3K, PERK, MAPK). They suppress pro-inflammatory kinases (PKA, mTOR, MAPKs), inhibit transcription factors (NF-κB, STAT3, AP-1), and downregulate inflammatory mediators (TNF-α, IL-1β, IL-6, COX-2). Curcumin also regulates the JAK/STAT pathway via SOCS1/3, blocks cyclin D1 and HER2-TK, and promotes antitumoral effects including cell cycle arrest, apoptosis, inhibition of tumor initiation and angiogenesis, and downregulation of adhesion molecules. Curcumin and its derivatives also exert chemosensitizing effects, in part through inhibition of thioredoxin reductase and restoration of an anti-inflammatory profile, further enhancing their antitumoral activity. These actions contribute to therapeutic potential in autoimmune diseases such as rheumatoid arthritis, multiple sclerosis, autoimmune thyroiditis, systemic lupus erythematosus, and uveitis.

*C. longa* is a rich source of structurally diverse bioactive compounds, among which the curcuminoids, such as curcumin, demethoxycurcumin, bisdemethoxycurcumin, and cyclocurcumin, are the most extensively studied group [33]. These polyphenolic pigments are primarily responsible for the plant’s characteristic yellow color and account for a substantial portion of its pharmacological activity. In addition to curcuminoids, *C. longa* contains other phenolic compounds such as ferulic acid and vanillic acid, as well as a complex mixture of essential oils comprising ar-turmerone, α-turmerone, and zingiberene, which contribute complementary biological effects [24]. Consequently, *C. longa* exhibits diverse pharmacological properties, including antioxidant, anti-inflammatory, antimicrobial, neuroprotective, and antitumor activities [31] (Figure 1).

Curcumin exerts its effects by modulating a variety of biological targets (Figure 1). Specifically, curcuminoids influence transcription factors like NF-κB and Nrf2, inflammatory enzymes such as COX-2 and iNOS, and cytokines including TNF-α, IL-1β, and IL-6, as well as signaling pathways related to cell proliferation and apoptosis like MAPK and PI3K/Akt [34]. Through these mechanisms, curcumin has demonstrated anti-inflammatory and pro-apoptotic effects in preclinical models.

Furthermore, curcumin impacts signaling cascades such as the JAK/STAT pathway, critical for cytokine-mediated signal transduction. This pathway relays extracellular polypeptide signals through specific receptors directly to nuclear target promoters, thus modulating gene transcription. Members of the JAK family participate in various inflammatory and autoimmune diseases [35]. Curcumin also modulates the activation of signal transducer and activator of transcription (STAT) proteins, which are vital for cell growth, differentiation, and survival, and play an essential role in the immune system’s development and functionality [36].

Moreover, curcumin has garnered attention for its ability to alleviate oxidative stress. It effectively neutralizes reactive oxygen species (ROS) and inhibits the formation of superoxide radicals, thereby protecting cells from oxidative damage. A pivotal mechanism through which curcumin exerts its effects is the activation of Nrf2, a crucial regulator of the body’s antioxidant response [37]. Activation of Nrf2 leads to an upregulation of cytoprotective proteins and bolsters the activity of antioxidant enzymes, thus enhancing the body’s defense against oxidative stress. Among these, curcumin has been shown to stimulate the expression of enzymes such as superoxide dismutase (SOD), catalase (CAT), and several kinases involved in cell proliferation and survival. Moreover, curcumin influences protein kinases like protein kinase C (PKC), phosphoinositide 3-kinase (PI3K), protein kinase RNA-like endoplasmic reticulum kinase (PERK), and mitogen-activated protein kinases (MAPK), which are integral in regulating various cellular functions, including proliferation and apoptosis. Additionally, curcumin can inhibit the activity of protein kinase A (PKA) and the mammalian target of rapamycin (mTOR), which are important regulators of metabolism and growth [38]. The JAK/STAT signaling pathway is intricately regulated by suppressor of cytokine signaling (SOCS) proteins, which play a pivotal role in modulating the production of pro-inflammatory mediators and cytokines [36]. Curcumin can inhibit lipopolysaccharide (LPS)-induced expression of crucial inflammatory cytokines such as IL-6 and TNF-α, as well as prostaglandin-endoperoxide synthase 2 mRNA in various cell models. This effect is attributed to curcumin’s ability to prevent the downregulation of SOCS1 and SOCS3, both of which are rapidly induced by cytokine stimulation and function by directly inhibiting JAK kinase activity [34,37]. Additionally, curcumin has been shown to attenuate inflammatory signaling by inhibiting LPS-induced phosphorylation of p38 MAPK, further suppressing pro-inflammatory cascades [37]. Beyond its effects on inflammation, curcumin also interferes with NF-κB signaling, which results in the suppression of cyclin D1 expression and activity. Cyclin D1 is a key regulator of the cell cycle and a transcriptional co-regulator whose elevated levels are often associated with cancer progression. Moreover, curcumin inhibits the transcription factor AP-1, known for its anti-apoptotic, pro-mitotic, and pro-angiogenic properties [38]. In the context of breast cancer, curcumin may have a particularly significant role, as it can inhibit the human epidermal growth factor receptor 2 (HER2-TK), both independently and in synergy with analogs. Given that the accumulation of HER2-TK is linked to the development of numerous cancers, targeting this pathway represents a crucial therapeutic strategy in oncology [19].

The multifaceted actions of curcumin are associated with antitumoral effects in vitro and in vivo, including mechanisms such as cell-cycle arrest, induction of apoptosis, and modulation of key signaling pathways. Intriguingly, curcumin’s influence on STAT3 may vary based on its concentration, while it can inhibit STAT3 phosphorylation in certain cancer contexts, it may promote an anti-inflammatory phenotype in vitro by enhancing STAT3 activity at lower concentrations [34,35]. These diverse mechanisms, associated with inflammatory and oncogenic pathways, highlight its potential as a promising adjuvant candidate for cancer prevention and therapy.

The growing scientific interest in curcumin-based preparations is supported by preclinical and clinical evidence highlighting the ability of *C. longa* to activate cellular immunity and modulate inflammatory processes, thereby acting as an immune enhancer [3]. Recent clinical and preclinical studies further reinforce curcumin’s immunomodulatory potential across a range of autoimmune and inflammatory diseases. Khamar et al. conducted a double-blind, randomized clinical trial evaluating Sina-curcumin, a nano micelle-based formulation, in patients with rheumatoid arthritis (RA). The curcumin group showed significant reductions in inflammatory markers (ESR and CRP) and a favorable shift in immune balance, marked by increased Th2 cell populations and a decreased Th1/Th2 ratio, supporting curcumin’s role as an adjunctive immunotherapeutic agent [39]. Yazdani et al. reported that curcumin exerts multifaceted effects in multiple sclerosis (MS), targeting mitochondrial dysfunction, apoptosis, autophagy, and signaling pathways such as AMPK, PGC-1α/PPARγ, and PI3K/Akt/mTOR [40]. These actions contribute to curcumin’s antioxidant and immunoregulatory properties, offering promise for future MS therapies. In an endocrine context, Papież demonstrated that curcumin reversed hypothyroidism-induced morphological and functional thyroid alterations in rats, improving follicular architecture, cytochrome c oxidase activity, and serum FT4 levels [41]. Allegri et al. showed that phospholipidic curcumin significantly reduced relapse frequency and severity in patients with recurrent anterior uveitis, with over 80% reporting symptom relief. This study also emphasized curcumin’s action as a PPAR-γ agonist, modulating both innate and adaptive immune responses [42]. Kotha and Luthria et al. studied the effects of curcumin as an anti-inflammatory agent in patients with head and neck squamous cell carcinoma and found that this phytochemical suppresses inflammatory cytokines such as TNF-α, IL-6, and IL-8 [38]. Collectively, these findings highlight curcumin’s broad immunomodulatory capacity and support its therapeutic relevance in diverse clinical settings.

In addition, 504 clinical trials are registered on ClinicalTrials.gov using the keywords “turmeric,” “curcuminoid,” or “Curcuma xanthorrhiza.” These trials are distributed across all continents, with the highest concentration in the USA and Asia; nevertheless, only 38 have published their results, and many involve relatively small sample sizes. Most focus on cancer treatments or inflammatory diseases. Among these, 27 are specifically related to curcumin, its derivatives, *C. longa* extracts, or fermented curcumin-based products, while the remaining 11 explore the effects of curcumin in combination with other compounds in specific pathologies. In the subset of published trials, curcumin has generally been well tolerated, with no clear signal of increased serious adverse events or treatment-related mortality compared with placebo; however, most studies are small, single-center, and of limited duration, and many are open-label or lack rigorous blinding, so conclusions about long-term safety and rare adverse events remain tentative [43]. Some studies have demonstrated the efficacy of curcuminoids in the treatment of oral lichen planus (OLP), a chronic mucocutaneous immune-mediated disorder. The first interim analysis did not show a significant difference between placebo and curcuminoid groups [44]. However, at higher doses of curcuminoids (6 g/day), patients showed greater reductions in erythema, ulceration, and the Modified Oral Mucositis Index, demonstrating efficacy in controlling OLP manifestations [45]. Taken together, clinical data on curcumin and its formulations are heterogeneous: some small, often single-center studies suggest symptomatic or biomarker improvements in specific indications, whereas others fail to show benefit over placebo, and robust evidence of disease-modifying effects from large, independently replicated, randomized controlled trials is still lacking. Karimi et al. evaluated the effects of curcumin nanomicelles (NC) on clinical outcomes and cellular immune responses in critically ill sepsis patients. NC supplementation decreased the expression of NLR family pyrin domain-containing protein 3 (NLRP3), interferon-gamma (IFN-γ), and NF-κB, as well as serum levels of IL-22 and IL-17 [46]. However, this study, like many others in the field, involved a relatively small cohort and has not yet been replicated, so the generalizability of these findings remains uncertain. Conversely, some clinical trials have failed to demonstrate beneficial effects of curcumin. Although in vivo studies suggest that curcumin can enhance the radioresponse in colorectal cancer, clinical results remain inconclusive. For instance, in patients receiving chemoradiation therapy (CRT) combined with curcumin (4 g twice daily), no significant improvement was observed in pathological complete response rates, indicating that promising preclinical results have not yet been consistently translated into clinical efficacy [47]. Similarly, Cruz-Correa et al. reported that 3 g/day of curcumin did not significantly affect the size or mean number of lower intestinal tract adenomas in patients with familial adenomatous polyposis [48]. Among users of the etonogestrel contraceptive implant who experienced frequent or prolonged bleeding, daily curcumin administration did not improve bleeding patterns [49]. Importantly, although curcumin has appeared safe in these short-term studies, systematic evaluation of long-term toxicity, drug–drug interactions, and the safety of high-dose or nano-formulations is still scarce, and publication bias in favor of positive or tolerability-focused studies cannot be excluded; thus, its overall safety profile should be considered provisional rather than definitively established.

The modest and inconsistent clinical efficacy observed to date likely reflects, at least in part, the poor and variable systemic exposure achieved with conventional oral preparations. The discrepancy between in vitro and in vivo studies and clinical trials is likely due to the unpredictable bioavailability of curcumin. Its low aqueous solubility, poor intestinal permeability, and rapid metabolic clearance result in poor bioavailability, which partly explains the persistent uncertainty surrounding its clinical efficacy [50]. This limitation was evident in rats receiving an oral dose of 1 g/kg; approximately 75% of the administered curcumin was excreted in feces, and serum concentrations remained extremely low or undetectable [51]. Consistent findings have also been reported in humans: despite the administration of high oral doses, circulating levels of curcumin and its metabolites remain minimal. After a single oral dose ranging from 500 to 8000 mg, no curcumin was detectable in serum at 1, 2, or 4 h after administration. Similarly, in patients with advanced colorectal cancer treated with 440–2200 mg/day of Curcuma extract for up to 29 days, neither curcumin nor its metabolites were detected in plasma or urine, underscoring the significant challenges associated with its systemic absorption [52]. These challenges limit the clinical impact of curcumin despite strong mechanistic evidence. Nevertheless, recent technological advances offer promising strategies to enhance their therapeutic potential.

## 5. *Echinacea purpurea* (L.) Moench

### 5.1. Ethnopharmacological Insights

*Echinacea purpurea* (L.) Moench, often known as the purple coneflower, holds a rich tradition in North American ethnomedicine, with documented use spanning several centuries among various Indigenous peoples. The term “Echinacea” encompasses nine plant species native to North America; specifically, *E. purpurea*, *Echinacea angustifolia* DC., and *Echinacea pallida* (Nutt.) Nutt. are the most commonly used.

Indigenous tribes, including the Cheyenne, Lakota, Dakota, Omaha, Pawnee, Ponca, Winnebago, and Kiowa, recognized Echinacea as a versatile remedy. They employed its roots, leaves, and flowers for a variety of therapeutic purposes, such as wound healing, alleviating fever, managing pain, treating toothaches, and addressing respiratory issues like coughs and colds [53]. This traditional knowledge reflects a holistic understanding of health, emphasizing the plant’s specific applications in relation to community health needs.

Among the Great Plains tribes, specific applications were highly detailed. Poultices made from crushed roots were commonly applied to burns, insect bites, ulcers, and infected tissue to reduce inflammation and promote healing. Decoctions and teas prepared from plant material were consumed to treat respiratory tract infections, persistent coughs, and to enhance physical endurance, especially during ceremonial rites such as sweat lodges. The Cheyenne and Kiowa chewed the roots to alleviate sore throats and thirst, while the Dakota, Omaha, and Pawnee used infusions and smoke treatments for headaches and systemic illnesses [53].

In contrast, the later eclectic medical interpretations of Echinacea often presented exaggerated claims, leading to its designation as a “panacea.” This modern usage promoted the herb as a treatment for an incredibly wide array of ailments, such as immune system disorders, sepsis, and tonsillitis, despite a lack of robust scientific support for such broad claims. Thus, while Indigenous uses were grounded in specific health contexts and demonstrated efficacy, the contemporary characterizations of Echinacea as a universal cure can lead to misconceptions about its effectiveness.

Indigenous knowledge was later incorporated into settler and modern herbal practices, leading to the widespread adoption of Echinacea and growing scientific interest in its immunomodulatory effects. Echinacea preparations were also regarded as general medicines used to strengthen the body’s resilience against disease and environmental stressors. This profound history not only demonstrates the plant’s pharmacological versatility but also underscores the significance of Indigenous knowledge in shaping contemporary understandings of its biological activity [53]. These ancestral practices significantly influenced its adoption into Western herbal medicine during the late 19th and early 20th centuries, when Echinacea was incorporated into the American eclectic medical tradition and promoted as a “blood purifier” and immune tonic [27].

From this rich ethnopharmacological background, *E. purpurea* entered modern phytotherapy and has since become one of the most widely used herbal medicines worldwide. The first commercial Echinacea product, known as Meyer’s Blood Purifier, was introduced around 1880. Commercial cultivation later began in Germany around 1939, and A. Vogel introduced and began cultivating the plant in Switzerland around 1950. This surge in popularity attracted the attention of chemists and pharmacologists, leading to the identification of numerous key constituents, including polysaccharides, echinacoside, cichoric acid, ketoalkenes, and alkylamides.

In contemporary contexts, *E. purpurea* is primarily recognized as a dietary supplement, widely marketed for the prevention and management of respiratory infections and to support immune function [54]. Its traditional roles in wound healing, infection management, and immune support provided the basis for current biomedical research and its widespread use in the prevention and treatment of upper respiratory tract infections, common colds, and influenza [27]. However, modern scientific evaluations have produced mixed outcomes due to variability in extract composition, preparation methods, and clinical endpoints [22].

### 5.2. Pharmacological and Immunomodulatory Profile

*E. purpurea* enhances the immune system through a complex mixture of bioactive compounds, including lipophilic alkylamides and ketoalkenes, alongside polar caffeic acid derivatives (e.g., cichoric acid, echinacoside), glycoproteins, and polysaccharides [55]. Manayi et al. (2015) reported that Echinacea exhibits immunomodulatory activity in preclinical models by stimulating the innate immune response, including the activation of macrophages and natural killer (NK) cells, which play critical roles in immune defense [56].

This activity aligns with the plant’s long-standing reputation as a medicinal species known for its potential benefits against colds and respiratory infections. Various in vivo and in vitro studies indicate that extracts from different parts of the plant, including roots and aerial parts, can stimulate immune responses [57]. However, the translation of these promising preclinical findings into consistent clinical efficacy has often been limited and requires further research to fully elucidate its significance in human subjects [22].

**Figure 2:**
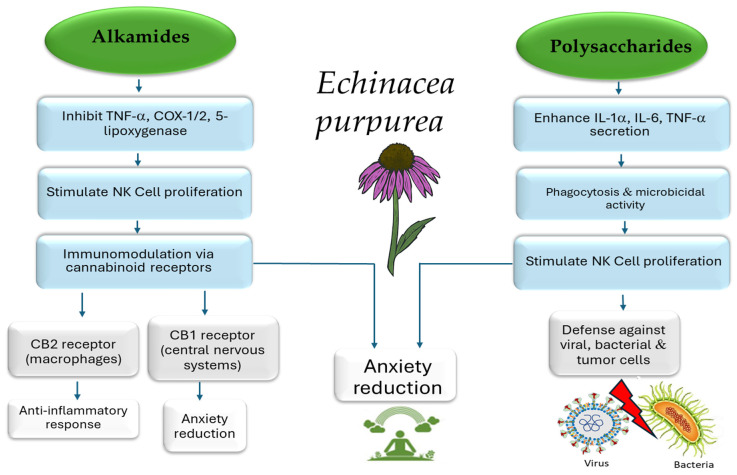
Conceptual schematic of the immunological and anti-inflammatory effects of *E. purpurea* metabolites. Alkamides and polysaccharides modulate cytokine profiles, NK cell activity, and cannabinoid receptor signaling, contributing to host defense and immune regulation.

One of the most studied classes is the alkamides. Figure 2 presents a schematic overview of some biological and pharmacological activities of alkamides isolated from Echinacea. Alkamides exert anti-inflammatory effects primarily by inhibiting key inflammatory enzymes, such as cyclooxygenase (COX) and 5-lipoxygenase (5-LOX), which are responsible for producing pro-inflammatory mediators like prostaglandins and leukotrienes [27,58]

Mechanistically, alkamides interact with cannabinoid receptor type 2 (CB2) expressed on macrophages, leading to various immunomodulatory effects [58]. They also stimulate the production of IL-10, a potent anti-inflammatory cytokine that helps suppress TNF-α expression [59]. Furthermore, alkamides have been shown to enhance NK cell responses in preclinical studies, potentially increasing their number and activity, which may improve defense against viruses and tumor cells [22].

Polysaccharides represent another major class of bioactive compounds, playing a pivotal role in immunomodulatory activity in preclinical models [60]. These molecules stimulate macrophages to produce pro-inflammatory cytokines such as IL-1α, IL-6, and TNF-α [60]. They also enhance phagocytosis and microbicidal functions, as demonstrated in both in vitro and in vivo studies [56]. Beyond macrophage activation and cytokine modulation, polysaccharide-enriched extracts promote the phenotypic and functional maturation of dendritic cells by modulating signaling pathways such as JNK, p38 MAPK, and NF-κB [55]. Additionally, they favor the polarization of M1 macrophages through modulation of the JAK pathway, collectively underscoring the complex, multi-target immunomodulatory and anti-inflammatory potential of Echinacea [61].

Tafazoli (2020) highlighted the potential of *E. purpurea* in oncology, sparking significant interest in its possible therapeutic applications [62]. Herbal complementary therapies have become increasingly popular among patients with cancer, with *E. purpurea* particularly favored by those with breast cancer [63,64]. The plant’s interaction with cannabinoid receptor 2 (CB2), which is overexpressed in breast tumor cells, supports its therapeutic relevance [63]. Research indicates that *E. purpurea* may suppress various cancers by increasing NK cell numbers and enhancing leukocyte phagocytic activity in the bloodstream. Among its active constituents, echinacoside stands out for its diverse biological effects, including neuroprotection, hepatoprotection, antiaging properties, immunomodulation, antidiabetic effects, and the promotion of bone formation. Most notably, the antioxidant properties of echinacoside underlie its neuroprotective and antitumor effects [65]. In vitro studies have shown that echinacoside selectively inhibits the proliferation of SW480 colorectal cancer cells in a dose-dependent manner, resulting in a reduction in colony number and size [64]. This inhibition is attributed to the ability of echinacoside to induce apoptosis by suppressing the enzyme MTH1, which normally protects cancer cells from oxidative DNA damage by hydrolyzing oxidized purines. This inhibition leads to increased oxidative DNA damage, upregulation of the G1/S-CDK inhibitor p21, and subsequent cell cycle arrest in the G1 and S phases, culminating in the selective apoptotic death of cancer cells without affecting healthy cells. Given the elevated production of reactive oxygen species (ROS) in tumor cells and their reliance on antioxidants, echinacoside effectively halts proliferation, induces apoptosis, and causes DNA damage specifically in cancer cells [64]. In addition, Aarland et al. (2017) assessed the biological properties of *E. purpurea* and *E. angustifolia*, focusing on their chemical composition and functional activities [66]. The study highlighted important pharmacological activities, including antioxidant, anti-inflammatory, hypoglycemic, and antiproliferative effects. The extracts, particularly those containing roots and aerial parts, showed promising antiproliferative effects against cancer cell lines and exhibited hypoglycemic activity comparable to standard drugs such as glibenclamide. The bioactive constituents responsible for these effects are believed to include alkamides, polysaccharides, glycoproteins, and phenolic compounds such as caffeic acid derivatives [66].

Clinical evidence also supports the immunomodulatory properties of *E. purpurea*. A randomized, double-blind, placebo-controlled clinical trial demonstrated the immunomodulatory effects of a 60% ethanolic extract of *E. purpurea* (EPE) in healthy Korean adults [67]. Participants were evaluated for NK cell cytotoxic activity, serum cytokine levels (IL-2, IL-6, IL-10, IL-12, IFN-γ, TNF-α), upper respiratory symptom scores (WURSS-21), and fatigue (MFS). After eight weeks of supplementation, EPE significantly enhanced NK cell activity and increased serum levels of IL-2, IFN-γ, and TNF-α compared with placebo, although no significant differences were observed in respiratory or fatigue scores. This was the first clinical trial to confirm the immune-enhancing potential of *E. purpurea* extract in Korean adults [67]. A total of 46 clinical trials were identified on ClinicalTrials.gov using the keywords Echinacea, echinacoside, or *Asteraceae*. Most trials were conducted in the United States and Europe (17 each), with additional studies registered in Asia and Africa; however, only six have published results to date. The majority addressed drug–herb interactions and immunological outcomes. Of these, four examined Echinacea, its derivatives, or Echinacea extracts, while two evaluated multi-herb combinations for selected pathologies [43]. In 2018, researchers at the University of Washington conducted two randomized controlled trials assessing the immunomodulatory effects of *E. purpurea*. In 2012, a three-arm RCT tested two commercially available preparations (5 mL or 1 mL orally three times daily for 10 days) versus placebo and found no significant differences in TNF-α, IL-6, IFN-γ, or IL-2 between groups [68]. A subsequent 2018 trial randomized 20 healthy adults to *E. purpurea* (100 mg/mL, 25 mL/day in two divided doses) or placebo for 10 days; blood samples collected before, during, and after treatment showed no meaningful changes in immune markers, suggesting minimal clinical effect [69]. Other randomized trials involving multi-herb formulations similarly failed to attribute significant benefits specifically to Echinacea. The Chinese University of Hong Kong (2010) tested a six-herb formula containing *Xanthium sibiricum* (*Asteraceae*) for perennial allergic rhinitis (1 g twice daily for 4 weeks) and observed no significant improvement in symptoms or quality of life; effects could not be attributed to Echinacea alone [70]. Pascoe Pharmazeutische Praeparate GmbH (2012) evaluated a homeopathic combination (Lymphdiaral Basistropfen, containing Echinacea and 11 other plant constituents) in chronic low back pain and reported significant symptom improvement versus placebo; however, the multi-component formulation precluded specific attribution of benefit to *Echinacea* [71].

Pharmacokinetic and physiological endpoints have also been investigated. The University of Georgia (2017) evaluated high-dose Echinacea supplementation (8000 or 16,000 mg/day) for effects on oxygen-carrying capacity and performance. Although erythropoietin (EPO) and interleukin-3 (IL-3) increased significantly at early time points (EPO: +44%, +63%, +36% at Days 7, 14, and 21; IL-3: +65% and +73% at Days 14 and 21), no significant changes were observed in red blood cell count, hemoglobin, hematocrit, VO_2_max, or anaerobic performance [72]. Similarly, the U.S. National Institutes of Health Clinical Center (2012) evaluated interactions between lopinavir/ritonavir and herbal products, including *E. purpurea* (500 mg three times daily for 14 days), and found no effect on lopinavir AUC, suggesting no clinically relevant pharmacokinetic interaction. However, participants in the Echinacea arm reported a higher incidence of mild gastrointestinal symptoms (diarrhea, nausea, abdominal discomfort) compared with those in the ginseng and ginkgo groups [73].

A systematic safety review by Ardjomand-Woelkart and Bauer (2015) summarized existing evidence regarding drug interactions, contraindications, and adverse events associated with Echinacea use, including during pregnancy and lactation [74]. They recommended caution in individuals with atopic conditions, given the unresolved potential for immune-related adverse effects. Across clinical studies conducted between 1996 and 2016, the most frequently reported side effects were mild headaches, dizziness, and gastrointestinal discomfort [74]. Consistently, Jeschke et al. (2009) conducted a large pharmacovigilance study in German primary care settings involving Asteraceae-based preparations, including Echinacea [75]. Among 18,830 patients who received 42,378 Asteraceae-containing remedies, 2672 were prescribed 4605 *Echinacea*-based products comprising 69 formulations. No serious adverse reactions were reported, and only 11 mild events occurred among 6961 prescriptions, supporting the conclusion that allergic or adverse responses to Echinacea products are rare and generally non-serious.

Overall, available randomized evidence indicates limited and inconsistent clinical benefits attributable specifically to Echinacea, with several trials reporting modest or transient immunological effects that do not consistently translate into meaningful outcomes.

Clinical evidence for *E. purpurea* remains heterogeneous and often limited, despite consistent immunological activity in preclinical models. Variability in extract composition, lack of standardization across commercial and experimental preparations, and substantial differences in study design contribute to the inconsistent results observed across clinical trials. Consequently, the strength of clinical evidence is considerably lower than that of mechanistic and observational data, underscoring the need for standardized preparations and well-designed trials capable of determining whether the immunomodulatory effects demonstrated experimentally can translate into meaningful clinical outcomes.

Beyond these clinical considerations, pharmacokinetic data provides additional insights into the behavior of *E. purpurea* constituents in humans. Human pharmacokinetic studies demonstrate that many alkamides from *E. purpurea* are systemically bioavailable after oral administration, with relatively rapid absorption, short Tmax, and measurable plasma concentrations, although Cmax and Tmax vary by formulation (tincture versus tablet). Evidence also suggests partial absorption via the oral mucosa. This systemic availability provides a plausible basis for immunomodulatory effects [56].

In contrast, polysaccharides, which are high-molecular-weight and water-soluble fractions of *E. purpurea*, exhibit immune-stimulating activity in both in vitro and in vivo models. These effects include macrophage and dendritic cell activation, cytokine induction, and enhancement of NK cell activity. Polysaccharides have also shown antiviral and anti-inflammatory protection in preclinical studies [57]. However, clinical evidence specific to polysaccharides remains inconclusive because most trials used whole-plant extracts containing multiple compound classes, making it difficult to isolate their individual contributions. Regulatory assessments likewise note heterogeneity in trial quality and extract composition.

The limited bioavailability of polysaccharides arises from their large molecular size and hydrophilicity, which restrict intestinal permeability and promote rapid degradation. Consequently, systemic effects observed in experimental models may depend on indirect mechanisms such as stimulation of gut-associated lymphoid tissue (GALT) or modulation of gut microbiota rather than high plasma concentrations [57]. This complicates pharmacokinetic-pharmacodynamic (PK-PD) interpretation.

In contrast, alkamides are more readily absorbed, but plasma concentrations vary considerably depending on extraction method, plant part, and dosage form (for example, tinctures or juices versus dried-root tablets). This variability contributes to inconsistent clinical outcomes among studies [56]. A major challenge remains the lack of standardization in commercial preparations, which are rarely quantified for specific alkamide or polysaccharide markers. The absence of such standardization reduces reproducibility and limits the comparability of results across trials, a point repeatedly emphasized in regulatory reviews as a barrier to firm clinical recommendations [62].

Certain Echinacea constituents have also been shown to influence cytochrome P450 enzyme activity in vitro, raising the possibility of herb-drug interactions. However, their clinical relevance appears limited and dependent on formulation, dosage, and duration of administration [62].

Beyond immunomodulation, Echinacea-derived phenylethanoid glycosides such as echinacoside have attracted attention for their antioxidant and cytoprotective properties. Echinacoside has been shown to induce apoptosis in cancer cell lines by promoting oxidative DNA damage and inhibiting nucleotide pool-sanitizing enzymes [60,76], as well as to delay cellular senescence in fibroblasts [65]. While echinacoside demonstrates robust antioxidant and cytoprotective effects in preclinical models (e.g., oxidative DNA damage induction in tumor cells, senescence delay in fibroblasts), no human clinical trials have demonstrated these antioxidant benefits; validated clinical outcomes remain limited to immunomodulatory effects on respiratory infections and NK cell activity.

## 6. Technological Innovations Enhancing Bioactivity and Bioavailability

The pharmacological potential of *Curcuma longa* L. and *Echinacea purpurea* (L.) Moench has been well established through decades of research, yet both species face significant biopharmaceutical limitations that hinder clinical translation. Poor aqueous solubility, chemical and metabolic instability, low intestinal absorption, and rapid clearance are key pharmacokinetic constraints [77]. These issues collectively limit systemic exposure and prevent the translation of promising preclinical findings into effective medicinal products. The variability observed across studies evaluating Echinacea and Curcuma-based preparations can be largely attributed to differences in extract formulation and phytochemical composition. Hydroalcoholic, aqueous, and standardized extracts differ markedly in their ability to solubilize and concentrate key secondary metabolites. Hydroalcoholic extracts typically yield higher levels of lipophilic and mid-polarity constituents such as alkylamides (in *E. purpurea*) and curcuminoids (in *C. longa*), which are often implicated in immunomodulatory and anti-inflammatory activity. In contrast, aqueous decoctions or infusions are enriched in hydrophilic components, including polysaccharides and glycoproteins, which produced a different immunological profile. Standardized extracts, although more consistent, vary depending on the specific marker compounds selected for standardization (e.g., total curcuminoids, alkamides, echinacoside), which may not fully represent the complexity of the native phytochemical matrix. As a result, cross-study comparison is inherently limited unless extraction methods and chemical fingerprints are explicitly reported. In addition, other factors contribute to the preparation variability and can explain the inconsistent clinical and experimental outcomes [3] such as differences in chemotype and Batch-to-batch variation. Both *Echinacea* spp. and *C. longa* display substantial genetic and chemotypic diversity. Variability in the relative abundance of alkylamides, caffeic acid derivatives, and polysaccharides in Echinacea, or curcuminoids and volatile terpenoids in Curcuma, leads to divergent biological activities. Without chemotype identification or chemical profiling, studies may inadvertently use botanically distinct materials under the same species name [75]. Environmental factors (soil, climate, harvest season), post-harvest handling, and extraction conditions can generate significant differences between production batches. Even in commercial preparations, variation in marker compounds can exceed acceptable limits unless rigorous quality control and authentication protocols are applied [78].

The nature of these limitations varies by compound class. For the curcuminoids from C. longa, the main metabolites responsible for immunomodulatory activity are highly lipophilic, poorly water-soluble, and extensively metabolized. This results in extremely minimal systemic availability even at high oral doses [79,80]. Overcoming this poor bioavailability is arguably the single greatest barrier to realizing curcumin’s full clinical potential. By contrast, the key immunomodulatory components of *E. purpurea* present a different set of challenges. Polysaccharides, for instance, are large, hydrophilic macromolecules with poor intestinal permeability and limited systemic absorption. While alkamides are generally better absorbed, their plasma levels are highly variable depending on the extraction method, specific plant part used, and the final dosage form [56,57,81]. Recognizing these constraints, the integration of pharmaceutical nanotechnology and formulation science has, in recent years, provided tangible solutions, giving rise to a new generation of standardized, technology-enhanced herbal preparations that bridge traditional use and modern pharmacotherapy [82].

Finally, the immunomodulatory effects of *C. longa* and *E. purpurea* depend on the specific immune parameters examined. Variability in the choice of biomarkers (e.g., cytokines, antibody titters, innate cell activity), timing of sampling, and heterogeneity in study populations can lead to disparate or non-comparable results. Furthermore, immune responses are highly context-dependent, and small methodological differences can shift outcomes from stimulation to suppression [3].

### 6.1. Advanced Nanodelivery Systems in C. longa

The therapeutic relevance of *C. longa* is attributed to its curcuminoids, including curcumin, demethoxycurcumin, and bisdemethoxycurcumin, which exhibit antioxidant, anti-inflammatory, and immunomodulatory activities [5,10]. Nevertheless, the clinical translation of these polyphenols has been hindered by major biopharmaceutical limitations, including poor water solubility (<0.1 mg/mL), chemical instability at physiological pH, rapid conjugation to glucuronides and sulfates, and low intestinal absorption leading to oral bioavailability below 1%. Most ingested curcumin is excreted in the feces, as reported in several animal studies [77]. These properties cause marked variability among studies and limit the translation of preclinical findings into reproducible clinical outcomes.

Curcumin has also been critically characterized in the pharmacological literature as a PAINS (pan-assay interference) and frequent-hitter compound. Numerous reported in vitro “activities” are now understood to arise from nonspecific reactivity, redox cycling, metal chelation, fluorescence interference, and aggregate formation rather than from selective binding to discrete molecular targets [83,84]. This phenomenon has contributed to an overextension of mechanistic claims across a broad range of pathways. Moreover, native curcumin is chemically unstable at physiological pH and undergoes rapid hydrolytic and oxidative degradation within minutes, generating multiple breakdown products with distinct pharmacodynamic profiles [85,86]. These instability kinetics offer a plausible explanation for the difficulty in reproducing mechanistic findings and the inconsistencies observed in clinical trials despite strong preclinical [25,87,88]. Recognizing these inherent limitations is essential for interpreting curcumin biology and for understanding why advanced formulation strategies are required to achieve translational reliability.

These methodological and chemical constraints also require a more cautious interpretation of the vast mechanistic landscape often attributed to curcumin. Many reports describe simultaneous modulation of diverse pathways, including NF-κB, Nrf2, JAK/STAT, MAPKs, PI3K/Akt, PERK, mTOR, and various receptor tyrosine kinases, yet only a subset of these interactions has been consistently reproduced under well-controlled conditions and at physiologically relevant concentrations. In particular, reports on STAT3 show predominantly inhibitory effects of curcumin, but with notable variability across cell types, inflammatory context, and concentration ranges, as well as by the interference phenomena inherent to PAINS-like behavior [89,90]. Consequently, mechanistic claims should prioritize pathways with strong and reproducible evidence, primarily those related to NF-κB suppression and Nrf2-dependent cytoprotective responses [86,91], while treating broader multitarget assertions with appropriate caution.

In light of these intrinsic molecular and analytical constraints, advanced formulation and nanodelivery strategies have been developed to improve curcumin’s stability, solubility, and translational reliability. These technologies aim to overcome the drawbacks of native curcumin and to optimize systemic exposure, tissue distribution, and pharmacodynamic performance.

Polymeric nanoparticles, solid lipid nanoparticles, nanoemulsions, liposomes, and cyclodextrin inclusion complexes are among the most investigated platforms because they enhance physicochemical stability, protect curcumin from degradation, improve aqueous dispersibility, and enable sustained release, resulting in higher systemic exposure compared with unformulated curcumin [80,81,82]. Encapsulation within cyclodextrins and biodegradable matrices has also improved solubility and stability under gastrointestinal conditions, supporting enhanced oral bioavailability [33,79,92].

Among plant-derived immunomodulators, *C. longa* stands as one of the most successful examples of nanotechnological advancement. Submicron colloidal dispersions such as Theracurmin^®^, a gum-ghatti-glycerin matrix dispersion, maintain curcumin in a stable colloidal form (<200 nm) and have shown approximately a 27-fold increase in AUC compared with unformulated curcumin [93,94]. Clinical trials have confirmed its safety and analgesic efficacy in knee osteoarthritis, reducing pain and the need for NSAIDs.

Building upon this formulation principle, subsequent advances have focused on further enhancing solubility and intestinal absorption through amorphization strategies. CurcuRouge™ is an amorphous solid-dispersion formulation of curcumin that enhances dissolution and absorption by transforming the crystalline compound into a stabilized amorphous state. Pharmacokinetic studies reported a 3.4-fold higher AUC and a 5.4-fold higher Cmax than Theracurmin^®^, demonstrating a consistent 3–5-fold improvement in systemic exposure [81,95]. Clinically, daily supplementation (180–720 mg) reduced inflammatory markers such as the neutrophil-to-lymphocyte ratio in elderly adults and tended to decrease fever incidence and antipyretic use in mild COVID-19, without safety concerns [96]. These findings confirm that amorphization improves bioavailability while preserving curcumin’s systemic anti-inflammatory and immunomodulatory effects.

A complementary approach involves nanomicellar encapsulation, which stabilizes curcuminoids within polymeric micelles, typically of approximately 10–20 nm. This marked improvement in dispersibility and intestinal uptake has led to significant advancements in bioavailability. SinaCurcumin^®^ (Exir Nano Sina, Tehran, Iran) is one of the best-characterized systems of this type, exhibiting up to sixty-fold higher bioavailability than unformulated curcumin.

Multiple randomized trials have reported consistent clinical and immunological benefits at low oral doses. For instance, in patients with osteoarthritis, a regimen of 80 mg day^−1^ for twelve weeks reduced pain and C-reactive protein. Immunologically, this treatment increased regulatory T cells (FOXP3^+^) while decreasing Th17 cells and BAFF-expressing B cells [97]. Similarly, in radiotherapy-induced oral mucositis, both oral (40 mg day^−1^) and topical 0.1% curcumin reduced ulcer severity and pain, with up to one-third of patients ulcer-free after three weeks [98].

Clinical efficacy has also been demonstrated in chronic and acute inflammatory conditions. In ulcerative colitis, 80 mg twice daily combined with mesalamine achieved higher clinical (63%) and endoscopic (89%) remission rates and reduced inflammatory markers like IL-17 and fecal calprotectin [99]. Furthermore, in the context of COVID-19, nanocurcumin at 160 mg day^−1^ accelerated the recovery of fever, cough, and dyspnea, and improved oxygenation [100], while outpatient trials showed faster symptom resolution and increased lymphocyte counts [101].

Mechanistic analyses across these studies consistently revealed a coherent immunological signature [39,102]. This signature is characterized by the suppression of Th17/IL-17-driven inflammation, together with the induction of regulatory networks mediated by IL-10, IL-35, and TGF-β, consistent with a beneficial shift toward immune tolerance and resolution. Overall, these findings demonstrate that advanced micellar systems can effectively translate pharmacokinetic optimization into clinically relevant immunoregulation across both chronic inflammatory and infectious diseases [39,97,99,100,101,102,103].

Beyond amorphous solid dispersions and nanomicelles, liposomal carriers have provided another effective strategy to overcome curcumin’s poor solubility and metabolic instability. Lipocurc^®^, a phosphatidylcholine-based liposomal formulation, has demonstrated superior plasma bioavailability and stability compared with unformulated curcumin. This design enables sustained systemic exposure and enhanced anti-inflammatory efficacy. Preclinical and early-phase clinical studies indicate favorable safety profiles and significant reductions in inflammatory mediators, positioning Lipocurc^®^ as a key intermediate in the evolution from colloidal to phospholipid-based delivery systems [104].

Specifically, a Phase I dose-escalation study was conducted in patients with advanced or metastatic cancer to establish safety and pharmacokinetics. The study showed that weekly intravenous infusions of 100–300 mg/m^2^ over 6–8 h were well tolerated, with 300 mg/m^2^ defined as the maximum tolerated dose. Curcumin plasma levels remained stable during infusion and declined rapidly afterward, with Cmax values around 1.4–1.6 µg/mL and near-linear increases in systemic exposure with the dose [105].

The liposomal formulation enabled consistent systemic delivery of both curcumin and its active metabolite, tetrahydrocurcumin (which accounted for ≈8% of the parent compound), notably without inducing hepatic or renal toxicity. Although no objective tumor responses were recorded, transient biomarker improvements suggested biological activity. These findings confirm that phospholipid encapsulation can provide sustained systemic exposure and pharmacological safety, establishing proof of concept for the translational potential of liposomal carriers for immune-relevant natural products [105].

Phytosomal complexes such as Meriva^®^, a curcumin-phosphatidylcholine conjugate, further improved bioavailability and metabolic stability. Clinical pharmacokinetic studies indicated a 29-fold increase in absorbed curcuminoids compared with unformulated mixtures [106]. In osteoarthritis, Meriva^®^ produced significant improvements in pain, stiffness, and physical function (WOMAC index) and reduced circulating interleukin-1β, interleukin-6, and soluble CD40 ligand [107,108]. Long-term trials also demonstrated benefits in diabetic microangiopathy, psoriasis, and inflammatory bowel disease, with improved microcirculation, retinal perfusion, and biomarker profiles [82,109,110,111].

Another clinically validated formulation, Flexofytol^®^, employs a micellar dispersion of curcumin stabilized with polysorbate surfactants to enhance dissolution and intestinal absorption [112]. Clinical trials in patients with osteoarthritis and tendinopathies have consistently shown significant pain reduction, improved joint function, and decreased reliance on nonsteroidal anti-inflammatory drugs, with no major adverse effects [113,114]. Although not a nanocarrier in the strict sense, Flexofytol^®^ demonstrates how micellar-based solubilization can yield both high bioavailability and robust clinical efficacy through optimized pharmaceutical engineering.

Lipid-based and micellar formulations such as Longvida^®^ and NovaSol^®^ represent additional translational advances. Longvida^®^, a solid-lipid curcumin particle, enhanced both acute and chronic cognitive performance in older adults while reducing serum cholesterol and demonstrating long-term tolerability [115]. NovaSol^®^ achieved an approximately 185-fold increase in AUC versus native curcumin, confirming the high solubilization potential of micellar carriers [116]. 

Parallel advances by Aquanova AG have produced liquid micellar systems employing polysorbate-based nanomicelles that achieve exceptionally high systemic exposure. In healthy volunteers, a single 500 mg dose of micellar curcuminoids yielded a 185-fold higher area under the plasma concentration-time curve and a Cmax exceeding 3000 nmol/L within one hour, compared with curcumin powder [116]. Subsequent studies showed that six weeks of daily supplementation (294 mg/day) led to steady-state plasma levels around 49 nmol/L without hepatic or renal toxicity, but no significant effects on serum lipids, C-reactive protein, or interleukin-6 in mildly hyperlipidemic subjects [117]. In a more recent double-blind crossover trial, micellar curcumin achieved Cmax and AUC values approximately 39- and 14-fold higher than native curcumin, respectively, yet failed to alter ex vivo interleukin-6 or TNF-α responses, with only a modest transient 10% reduction in PCSK9 not reproduced in larger cohorts [118]. These findings underscore that enhanced pharmacokinetic exposure alone does not necessarily translate into immunological or metabolic efficacy in vivo, emphasizing the need to align technological optimization with biological relevance.

Recent developments extend these concepts toward microfluidic phospholipid nanosuspensions that yield uniform particles of approximately 150 nm with improved physicochemical stability, and liposomal–polymeric hybrids that combine lipid bilayers with biodegradable polymer coatings for controlled release [80,119]. Together, these innovations illustrate how phospholipid-based carriers emulate natural membrane interactions to achieve biomimetic pharmacokinetics, stabilizing plasma concentrations of labile plant metabolites and sustaining systemic immunomodulatory effects through regulation of NF-κB, interleukin-6, and interleukin-22. This convergence of stability, absorption efficiency, and immune modulation positions liposomal, phytosomal, and micellar systems as critical transitional stages in the evolution of technologically enhanced, bioinspired phytopharmaceuticals.

An alternative strategy to overcome the poor bioavailability of herbal metabolites is the use of bioenhancers, compounds that increase the absorption and systemic exposure of co-administered phytochemicals. The best-known example is piperine, an alkaloid from *Piper nigrum*, which inhibits glucuronidation and intestinal efflux transporters, thereby significantly enhancing plasma concentrations of curcuminoids in humans. Recent clinical trials have demonstrated that improved curcumin formulations (for example, curcumin plus piperine or curcumin nanomicelles) can modulate inflammatory cytokines and immune parameters in various conditions, from exercise-induced inflammation to sepsis and viral infections. These findings support the concept that increased systemic exposure translates into measurable immunomodulatory outcomes [46,120,121]. 

Beyond nanocarriers, co-formulations with natural bioenhancers such as piperine have proven effective at inhibiting hepatic and intestinal glucuronidation, further increasing curcuminoid plasma concentrations. Encapsulation in cyclodextrins and biodegradable biopolymers has also conferred protection against oxidative degradation and enabled controlled release under simulated gastrointestinal conditions [9,10].

Technological advances in phytopharmaceutical formulation have focused not only on enhancing systemic concentrations but also on achieving targeted and responsive delivery of bioactive compounds. In the case of curcuminoids, pH-sensitive nanocarriers have been developed to release their payload preferentially within inflamed or diseased tissues, where acidic microenvironments prevail. Likewise, ligand-functionalized nanoparticles enable selective uptake by macrophages or dendritic cells, optimizing curcumin’s immunomodulatory action and minimizing off-target effects [122,123]. These approaches are particularly relevant in immune-mediated disorders or localized inflammation, where therapeutic precision and reduced systemic exposure are critical. Beyond nanoparticles, hydrogels and scaffold-like biomaterials have emerged as promising platforms for localized and sustained curcumin release. Hydrogels can be engineered to respond to environmental stimuli such as pH, temperature, or enzymatic activity, enabling controlled release in wound or tissue repair contexts. Recent studies have demonstrated that curcumin-loaded hydrogels effectively modulate inflammatory cytokines, accelerate wound closure, and promote tissue regeneration [124]. This class of materials exemplifies the transition toward multifunctional biomaterials that simultaneously deliver phytochemicals and support regenerative processes.

Overall, the evidence summarized in Table 2 indicates that *C. longa* has reached an advanced level of pharmaceutical translation. These data confirm that *C. longa* has successfully progressed from laboratory formulations to well-characterized clinical nanocarriers. Its various nanocarrier systems consistently enhance bioavailability, attenuate inflammatory biomarkers, and maintain excellent safety across multiple clinical contexts, including arthritis, metabolic disorders, and systemic inflammation. The cumulative data establish *C. longa* as a benchmark phytopharmaceutical model, providing both technological and regulatory guidance for future development of other plant-derived immunomodulators such as *Echinacea purpurea*, discussed in the following subsection.

### 6.2. Pharmaceutical and Nanotechnological Innovations in E. purpurea

Following the advanced nanodelivery developments observed in *C. longa*, *E. purpurea* remains at an earlier translational stage. The main challenge for the pharmacological potential of *Echinacea* lies in the systemic availability of its polysaccharides, as these compounds are minimally absorbed. Experimentally observed systemic effects are often attributed to indirect pathways such as activation of gut-associated lymphoid tissue (GALT) or stimulation of gut microbiota, rather than measurable plasma levels [57]. These characteristics make it difficult to clearly link pharmacokinetic behavior with pharmacodynamic responses in the case of polysaccharides. In contrast, alkamides exhibit a much better absorption profile, although their circulating concentrations vary considerably depending on the extraction procedure, the plant part used, and the dosage form [56].

Systematic reviews and meta-analyses suggest consistently support the clinical efficacy of *E. purpurea* extracts in preventing and mitigating viral respiratory tract infections. Evidence from controlled trials and pooled analyses shows that standardized preparations can reduce the incidence, duration, and recurrence of common colds and other respiratory infections, with a favorable safety profile [54,131,132]. These findings highlight the therapeutic potential of *Echinacea* as an immunonutritional strategy for respiratory health. However, the interpretation of clinical evidence for Echinacea is complicated by significant methodological and commercial heterogeneity, which must be clearly acknowledged. Systematic reviews, while often suggesting overall benefit, consistently note high methodological heterogeneity across trials, including differences in species (*E. purpurea* vs. *E. angustifolia*), plant parts, and extraction conditions, which complicates direct comparison and synthesis of results [54,133]. Several independent randomized trials have reported neutral outcomes, particularly when using non-standardized or less bioavailable preparations. For example, *E. angustifolia* root extract showed no significant benefit in reducing cold duration or severity [134], and pressed juice from *E. purpurea* failed to improve symptom scores compared to placebo [135]. These findings reinforce the importance of formulation-specific validation and caution against generalizing efficacy claims across the diverse Echinacea product landscape. Furthermore, the Echinacea market is highly heterogeneous, critical analyses confirm that the wide array of commercial preparations often differs significantly from the specific standardized preparations used in definitive clinical studies, limiting the generalizability of positive findings to the wider commercial landscape [136].

Technological development initially focused on the standardization of hydroethanolic extracts rather than nano-delivery systems. Consequently, most human clinical trials have employed a limited number of specific, standardized preparations, primarily the 65% hydroethanolic preparation, marketed as Echinaforce^®^ (EF), which contains a fixed ratio of herb and root extracts rich in alkamides and caffeic acid derivatives [130]. EF, a standardized hydroethanolic extract derived from freshly harvested aerial parts and roots of *E. purpurea*, was developed to optimize the preservation and bioavailability of its key bioactive constituents, including N-alkylamides, caffeic acid derivatives (particularly cichoric acid), and polysaccharides. Pharmacokinetic investigations have shown that oral administration of EF yields detectable plasma levels of dodeca-2E,4E,8Z,10E/Z-tetraenoic acid isobutylamides within 30 min, with a Tmax of approximately 1 h and a short elimination half-life, indicating efficient intestinal absorption and systemic exposure of lipophilic alkamides [137]. These findings support the role of alkylamides as primary contributors to the immunomodulatory effects of *E. purpurea*, acting through partial agonism of the CB_2_ cannabinoid receptor and modulation of cytokine production in monocytes and macrophages [138]

In prevention trials, EF reduced infections with enveloped viruses, including coronaviruses and SARS-CoV-2, by up to 60%, lowered viral loads, and accelerated recovery, all with excellent tolerability [130]. Pediatric formulations such as EF Junior decreased respiratory tract infection (RTI) frequency and antibiotic use by more than 70% [139]. High-dose EF lozenges and sprays improved viral clearance during acute infection [127], and meta-analytical evidence encompassing more than 5000 participants confirmed significant reductions in monthly RTI incidence (RR ≈ 0.68), recurrence, and complication rates, with no increase in adverse events [54,131,132]. Subsequent cell-based studies confirmed that the combination of alkamides and caffeic acid derivatives exerts synergistic effects on macrophage activation and antiviral gene expression, supporting the rationale for preserving the native phytochemical spectrum in whole-plant formulations [140]. Beyond immune health, *Echinacea* preparations have also been investigated for potential benefits in inflammatory and stress-related conditions. Intake of *Echinacea* extracts may attenuate the mucosal immune suppression that occurs during intense exercise. In a study involving 32 subjects performing an exercise protocol known to affect mucosal immunity, supplementation reduced the duration of upper respiratory tract infections (URTI) [141]. These findings establish *E. purpurea* as one of the most pharmacologically characterized immunomodulatory botanicals, whose standardized extracts, particularly EF, exhibit reproducible pharmacokinetics, a favorable safety profile, and clinically relevant benefits in the prevention and mitigation of respiratory infections and inflammatory stress.

Beyond these products, recent years have witnessed a rapid emergence of preclinical nanotechnological approaches aimed at enhancing the pharmacological performance of *Echinacea*. While *E. purpurea* has achieved clinical validation through standardized extracts such as EF, its nanotechnological development remains at a much earlier, primarily preclinical stage. Most nanoformulation studies are exploratory and have not yet progressed to human trials. It is therefore important to distinguish between the translational maturity of clinically tested standardized extracts and the experimental status of emerging nanocarrier systems. This review treats these domains separately to avoid conflating their levels of validation.

Experimental systems include polymer-inorganic hybrid nanoparticles such as chitosan-silica (CSE) nanocarriers [142], which improved antioxidant defenses and reproductive protection in vivo; phosphatidylcholine liposomes embedding phenolic-rich extracts [132], achieving approximately 78% encapsulation and sustained 24 h release; and several “green-synthesized” metallic nanoparticles (AgNPs, Fe_2_O_3_) that combine strong antioxidant and antibacterial activities with demonstrated cytocompatibility [143,144,145]. Additional innovations include electrosprayed polymeric nanoparticles [146] that stimulated immune activation in vivo without toxicity, and electrospun keratin nanofibers incorporating *Echinacea* extract and biosynthesized AgNPs [147], which showed potent antimicrobial and wound-healing properties. 

The technological advances achieved in the targeted and responsive delivery of curcuminoids provide a valuable framework for improving the pharmacological translation of *E. purpurea* extracts. Similar to curcumin, the major bioactive constituents of *E. purpurea*, particularly alkamides and polysaccharides, face challenges related to low and variable bioavailability. For example, alkamides in *E. purpurea* have demonstrated rapid degradation under storage conditions, indicating stability issues [148].

Nanocarrier systems such as liposomes and lipid-based nanosystems have already been applied in *E. purpurea*, for instance in topical gels incorporating liposome- or transferosome-entrapped extracts, which showed improved release profiles and preserved antioxidant activity. Empirical work demonstrates feasibility in *E. purpurea*: lipid-based nanosystems (liposomes and transferosomes) loaded with *E. purpurea* extract achieved nanometric sizes (approximately 156–199 nm after loading), high entrapment efficiencies (about 63–75%), and improved biocompatibility and sustained release when incorporated into topical gels [149].

Following this paradigm, polymeric nanoparticles (for example, chitosan-silica nanoparticles loaded with *E. purpurea* extract, approximately 145 nm in diameter) have been tested in vivo, showing enhanced anti-inflammatory effects compared with non-encapsulated extracts [150]. Electrospray-fabricated polymeric nanoparticles (for example, Eudragit RS100) have also been used to encapsulate *E. purpurea* extract, demonstrating that particle engineering can modify immunomodulatory outcomes in vivo [146]. Even green-synthesized inorganic nanoparticles derived from *E. purpurea* (hematite, silver) have shown altered antimicrobial, antioxidant, and cytotoxic profiles compared with crude extract, illustrating that nano-structuring the botanical matrix can meaningfully modify bioactivity [145]. 

Therefore, nanoemulsions, liposomes, polymeric nanoparticles, or even more advanced responsive carriers such as pH- or enzyme-sensitive systems or magnetic and photo-activated platforms could be engineered to enhance alkamide absorption, prolong systemic or local exposure, protect labile polysaccharides from gastrointestinal degradation, and allow targeted release into GALT or immune-cell compartments [144]. These approaches not only address pharmacokinetic limitations but also open new avenues for precise immune modulation, positioning *E. purpurea* as a candidate for next-generation phytopharmaceutical formulations.

Applied to *E. purpurea*, this concept suggests that combining alkamide-rich extracts (lipophilic compounds with favorable oral absorption) with bioenhancers or advanced formulation strategies (such as nanoemulsions or lipid complexes) could improve systemic availability and thereby potentiate immunomodulatory effects. Clinical studies involving curcuminoid bioenhancement show that co-administration with piperine or formulation with other polyphenols (for example, curcumin plus quercetin) and fatty acids can reduce pro-inflammatory cytokines (IL-6, TNF-α) and oxidative stress markers, providing proof of concept for similar formulation strategies in herbal immunomodulators like *Echinacea* [17,121].

However, clinical implementation of bioenhancers in *Echinacea* preparations must be approached with caution. Inhibition of metabolic pathways (UGT, CYP) or efflux transporters could increase the risk of herb-drug interactions or alter the pharmacokinetics of concomitant medications. Moreover, variability in *Echinacea* extract composition and the lack of standardization of marker compounds (for example, alkamides and polysaccharides) complicate extrapolation from curcumin studies to *Echinacea*. Therefore, before recommending enhanced formulations for routine use, specific pharmacokinetic studies (Echinacea plus bioenhancer) and controlled clinical trials measuring both systemic exposure and immune outcomes are needed, along with careful safety and interaction evaluations [46,120]

Because most of these formulation strategies remain at the preclinical or early translational stage, their significance must be interpreted relative to the available human evidence for *E. purpurea*, which is derived primarily from standardized herbal preparations. This body of clinical data provides the reference framework for assessing newer formulation approaches. At present, nanotechnological and experimental delivery systems should be regarded as exploratory strategies aimed at addressing pharmacokinetic limitations rather than as clinically established interventions and are summarized in Table 3.

## 7. Comparative Analysis Between *Curcuma longa* L. and *Echinacea purpurea* (L.) Moench

Although *Curcuma longa* L. *and Echinacea purpurea* (L.) Moench are botanically distinct species with different ethnomedical origins, namely Asian Ayurvedic medicine and North American herbalism, they converge pharmacologically as plant-derived immunomodulators with well-characterized anti-inflammatory and immune-balancing properties [3,18]. Both species exert multilevel regulation of immune pathways through bioactive compounds that act on transcriptional and signaling cascades, modulating the crosstalk between innate and adaptive immunity [3,18].

The immunomodulatory actions of both *C. longa* and *E. purpurea* share a core mechanism centered on the inhibition of NF-κB, MAPK, and JAK/STAT pathways, which are key regulators of inflammatory cytokine production [5,10]. Their bioactive constituents, mainly curcuminoids in *C. longa* and alkylamides together with caffeic acid derivatives in *E. purpurea*, influence multiple molecular routes that converge in the suppression of pro-inflammatory mediators such as IL-1β, IL-6, and TNF-α. Simultaneously, they enhance antioxidant and cytoprotective defenses mediated by Nrf2 and heme oxygenase-1 [5,10].

Both plants also regulate the arachidonic acid cascade through modulation of COX activity, a central mechanism in prostaglandin synthesis and inflammation control. However, they achieve this differently. Curcuminoids from *C. longa* directly downregulate COX-2 expression and inhibit iNOS, leading to decreased prostaglandin and nitric oxide production. In contrast, alkylamides from *E. purpurea* primarily act by partially inhibiting both COX and LOX enzymes, thereby limiting the formation of prostaglandins and leukotrienes [3,18,46]. These shared, yet distinct, actions attenuate inflammatory mediator synthesis and illustrate their complementary regulation of enzymatic and cytokine-driven inflammation (IL-2, IL-6, TNF-α), reinforcing their adaptogenic immunomodulatory capacity.

*E. purpurea* further demonstrates distinctive molecular interactions critical for innate immunity and antiviral defense. These include the activation of Toll-like receptors TLR2 and TLR4 and the unique modulation of the cannabinoid receptor type 2 (CB2) [13,14]. These pathways collectively enhance macrophage and natural killer (NK) cell activity, highlighting mechanisms that initiate innate immune responses and are crucial for the plant’s established role in antiviral defense.

Based on the current clinical literature, there is stronger human evidence supporting acute immunomodulatory effects for Echinacea, particularly in the prevention and early treatment of upper respiratory tract infections (URTI), than for chronic immune-related disorders. Multiple randomized controlled trials and systematic reviews, including the Cochrane review, suggest modest but measurable reductions in the incidence or duration of URTIs, although the magnitude of the effect varies across studies due to heterogeneity in extract type, dosage, and phytochemical composition [132].

In contrast, the most consistent human evidence for *C. longa* (curcuminoids) relates to its role in chronic inflammatory conditions rather than acute immunomodulatory responses. Studies demonstrate reductions in systemic inflammatory markers (e.g., CRP, TNF-α, IL-6) and improvements in symptoms in disorders such as rheumatoid arthritis, metabolic syndrome, and inflammatory bowel disease. However, evidence for curcumin in acute infections or short-term immune activation in humans remains limited, partly due to its inherently low oral bioavailability and the need for enhanced formulations to achieve physiologically relevant plasma levels.

Each species exhibits a distinct therapeutic emphasis according to its phytochemical composition and primary clinical application. The findings support the functional difference between the two. *C. longa* acts primarily as a broad-spectrum anti-inflammatory and potent antioxidant regulator, effective against chronic, low-grade inflammation. *E. purpurea*, conversely, serves as an immunostimulant that optimizes innate immunity and antiviral activity, reinforcing host defense, particularly under conditions of transient immune suppression or viral infection. Both species, therefore, complement each other pharmacodynamically. However, the clinical translation of these established benefits remains severely limited by pharmacokinetic constraints. For curcuminoids, the challenge is clear: poor aqueous solubility, low gastrointestinal absorption, and rapid metabolism result in minimal systemic bioavailability, necessitating the use of advanced formulations [5,9,10]. For *E. purpurea*, the primary limitation is often standardization. Preparations display significant variability in alkylamide and phenolic acid concentrations depending on the specific plant part, the extraction solvent, and cultivation conditions, leading directly to inconsistent biological activity in trials [3,10]. Recent technological advances have sought to address these distinct limitations. In the case of *C. longa*, nanoformulations such as phytosomes, liposomes, nanoemulsions, and co-formulations with bioenhancers like piperine have significantly improved solubility, absorption, and pharmacological stability [5,9,10]. Although fewer technological studies are available for *E. purpurea*, encapsulation in lipid or polymeric matrices and the use of green extraction methods have shown potential for preserving bioactive integrity and enhancing absorption. This places both species within the same framework of modern pharmaceutical innovation. A comparative summary of their principal pharmacological and technological features is presented in Table 4, which emphasizes their complementary immunomodulatory profiles and formulation advances.

Overall, *C. longa* and *E. purpurea* demonstrate a high degree of mechanistic and technological complementarity. The anti-inflammatory and antioxidant actions of curcuminoids can mitigate chronic inflammation and oxidative stress, while the immunostimulant and antiviral properties of *E. purpurea* can reinforce the host’s first-line defense. Their combined use could therefore promote a balanced immune response that attenuates excessive inflammation without compromising immune vigilance, a concept consistent with adaptive immunoregulation [18,19]. Furthermore, their compatibility with nanotechnological and biotechnological formulation strategies positions these two species as complementary candidates for the design of next-generation plant-based immunotherapeutics. In this sense, *C. longa* and *E. purpurea* represent not only parallel models of phytochemical immunomodulation but also synergistic systems capable of integrating traditional knowledge with contemporary pharmaceutical science.

## 8. Future Perspectives and Translational Potential

The growing body of evidence supporting the immunomodulatory potential of *Curcuma longa* L. and *Echinacea purpurea* (L.) Moench highlights a promising pathway for translating traditional herbal pharmacology into standardized, evidence-based immunotherapeutics and nutraceuticals [3,18]. Despite strong preclinical and clinical support, several barriers still limit their full translation, including variability in phytochemical composition, differences in extraction methods, and the inherently low bioavailability of their active compounds [5,10]. Overcoming these challenges will require integrative strategies that combine pharmacological, biotechnological, and regulatory innovation to ensure consistency, safety, and reproducibility. Recent advances in nanotechnology and sustainable pharmaceutical design have already improved the solubility, stability, and targeted delivery of phytochemicals such as curcuminoids and alkylamides. Future research should expand these approaches to large-scale, clinically validated formulations that meet regulatory and good manufacturing practice standards [5,9,10]. Moreover, the integration of *omics*-based tools, metabolomics, transcriptomics, proteomics, and network pharmacology, combined with artificial intelligence and computational modeling can accelerate mechanism discovery, biomarker identification, and patient stratification, facilitating personalized immunonutrition strategies and data-driven product optimization.

From a translational standpoint, the complementary mechanisms of *C. longa* and *E. purpurea*, anti-inflammatory and antioxidant regulation by curcuminoids, and innate immune activation and antiviral support by alkylamides, position them as attractive candidates for functional foods or immunonutritional interventions aimed at post-viral recovery and chronic low-grade inflammation [18,19]. Their adaptogenic balance could also be explored for applications in veterinary immunomodulation or as plant-based bioinputs designed to enhance animal health and resilience, representing a bridge between biomedical and agro-biotechnological innovation.

At the regulatory level, harmonizing quality control, standardization, and labeling frameworks across herbal and nanoformulated products remains an urgent priority. Establishing global benchmarks for bioactive quantification, safety testing, and clinical evidence would facilitate approval processes and encourage the integration of phytopharmaceuticals into public health strategies. Collaborative efforts between academia, industry, and health agencies will be critical to ensure that innovation proceeds in a safe, traceable, and sustainable manner [9]. In line with the World Health Organization’s support for integrating traditional medicine into primary healthcare (WHO, 1991), and in accordance with the nursing code of ethics (ICN, 2021), nurse researchers, together with other health professionals, should rigorously evaluate these innovations through clinical trials to ensure their safe and effective implementation.

Future research on Echinacea and Curcuma-derived products should focus on strategies that reduce variability and strengthen mechanistic understanding to enable more reliable clinical translation. Several emerging directions are particularly promising.

(1) AI-driven optimization of botanical formulations.

Advances in machine learning offer new opportunities to improve formulation consistency and bioavailability. AI-based models capable of integrating phytochemical profiles, pharmacokinetic parameters, and biological activity could help identify optimal combinations of bioactive constituents, predict synergistic interactions, and support the design of standardized extracts with enhanced functional properties.

(2) Multi-omics approaches to define response phenotypes.

Applying integrative multi-omics, including metabolomics, transcriptomics, proteomics, and advanced immunophenotyping, may help clarify the heterogeneity of immunomodulatory responses seen in human studies. These approaches can aid in identifying responder and non-responder subgroups, distinguishing acute versus chronic immunological signatures, and linking specific phytochemical patterns to defined biological pathways. Such insights would improve the precision and reproducibility of future clinical trials.

(3) Regulatory frameworks for botanical nanoformulations.

The increasing use of nano-delivery systems to improve the solubility and stability of curcuminoids and other lipophilic constituents highlights the need for clear regulatory guidance. Standardized requirements for characterization, quality control, safety assessment, and labeling of nanoformulated botanical products would support harmonization across studies and facilitate their safe translation into clinical use.

Overall, these future directions underscore the importance of integrating technological innovation, systems biology, and regulatory development to enhance the translational potential of botanical immunomodulators.

## 9. Conclusions

*Curcuma longa* L. and *Echinacea purpurea* (L.) Moench contain metabolites with documented immunomodulatory activity in preclinical models. Evidence for *C. longa* is comparatively stronger, supported by extensive mechanistic research and several clinical investigations, including studies evaluating advanced formulations with improved pharmacokinetic performance. In contrast, although *E. purpurea* shows consistent immunological activity in vitro and in vivo, clinical outcomes remain variable and often limited in magnitude.

For both species, translation from preclinical findings to clinical benefit is hindered by low and inconsistent oral bioavailability, rapid metabolism, and substantial variability in extract composition across commercial and experimental preparations. These factors contribute to heterogeneous study outcomes and limit reproducibility. Advances in formulation science, including micelles, phytosomes, nanoemulsions, polymeric nanoparticles, and liposomal systems, have improved solubility and systemic exposure of key metabolites and have generated preliminary biological and clinical signals.

Overall, current evidence suggests that *C. longa* and *E. purpurea* possess immunomodulatory potential, but existing clinical data remain insufficient to support therapeutic claims. Progress will require standardized preparations, rigorous phytochemical characterization, and well-designed clinical trials capable of linking specific formulations to defined immunological and clinical endpoints. The integration of pharmacological insight with formulation technology will be essential to determine the real clinical value of these species within evidence-based immunomodulatory strategies.

## Figures and Tables

**Table 2:** Representative human clinical studies evaluating advanced curcumin delivery systems. Formulations are listed in approximate chronological and technological order, from early nanoparticle colloidal dispersions to amorphous solid dispersions, phosphatidylcholine phytosomes, micellar/microemulsion systems, and finally liposomal intravenous formulations. This organization reflects the progressive optimization of curcumin bioavailability and clinical applicability. Proprietary formulation names are reported solely for identification as provided in the original studies and do not imply endorsement; emphasis is placed on the underlying nanotechnological platform and study design rather than brand identity.

Formulation Type	Study Design	Population/Condition	Sample Size/Dose	Endpoint Focus	Key Findings	Ref.
Submicron colloidal curcumin (Theracurmin-type)	RCT, open-label PK + exploratory efficacy	Healthy volunteers; osteoarthritis	*n* = 50 (OA) (PK)/30–210 mg/day oral	PK + pain scores	25–30× higher bioavailability; improved knee pain/function; only mild gastrointestinal or skin adverse effects reported.	[93,125,126,127]
Polymer-stabilized/Amorphous solid dispersion (CurcuRouge-type)	Randomized, double-blind	Elderly volunteers; mild COVID-19	*n* = 39 (elderly), *n* = 145 (COVID)/90–720 mg/day oral	Immunological endpoints (NLR, cytokines)	Reduced neutrophil-to-lymphocyte ratio; fewer COVID-19 progression events; safe. CurcuRouge™ showed higher bioavailability and faster absorption than Theracurmin^®^, with no safety concerns.	[81,128,129]
Phosphatidylcholine phytosome (Meriva-type)	Randomized, double-blind; open-label controlled	Osteoarthritis, psoriasis, diabetes microangiopathy, pancreatic cancer	*n* = 63 (psoriasis), *n* = 77 (diabetes), *n* = 100 (OA), *n* = 44 (cancer)/2000–500 mg/day	Inflammatory markers (IL-1β, IL-6, IL-22, CRP)	Significant reduction in pain and improved physical function (WOMAC, VAS); decreased IL-1β, IL-6, IL-22, CRP, sCD40L, ESR; improved microcirculation and edema in diabetes; in pancreatic cancer, 61.4% disease control rate and 10.2-month overall survival; quality of life maintained; no serious adverse events	[106,108,109,110,111]
Micellar curcumin (NovaSol-type)	Randomized, crossover PK + efficacy	Healthy adults; hyperlipidemia	*n* = 42 (lipid trial), *n* = 15 (immune endpoints)/129–500 mg/day	PK + exploratory immune markers	Up to 453× higher Cmax and 185× higher AUC vs. native curcumin; faster absorption (Tmax ≈ 1.1 h); sex differences favoring women; no significant changes in IL-6, TNF-α, CRP, or lipid profile; plasma PCSK9 reduced ~10%; well tolerated with mild GI symptoms	[116,117,118]
Micellar microemulsion curcumin (Flexofytol-type)	Observational; open-label; RCT	Osteoarthritis (various joints; knee KL II–III)	*n* = 820 (real-life), *n* = 22 (exploratory), *n* = 150 (RCT)/168–252 mg/day oral (4–6 caps/day)	Pain, function, biomarkers (Coll2-1, CRP)	Significant pain reduction and improved joint function; decreased Coll2-1 and CRP; reduced NSAID/paracetamol use; quality-of-life gains; well tolerated with mild GI events; real-life benefit confirmed in RCT	[112,113,114]
Nano-micellar curcumin (SinaCurcumin-type)	Randomized, double-blind	COVID-19, rheumatoid arthritis, ulcerative colitis, osteoarthritis	*n* = 30–40 per trial/40–160 mg/day oral	Cytokines (IL-17, IL-4, CRP, ESR, miRNA)	Improved CRP/ESR; cytokine modulation; clinical benefit in UC and RA	[39,97,99,100,103]
Liposomal intravenous curcumin (Lipocurc-type)	Phase I, open-label	Advanced solid tumors	*n* = 32/100–300 mg/m^2^ IV	PK + safety	Steady plasma levels during infusion; safe up to 300 mg/m^2^. Tumor marker changes; transient PSA/CEA/CA19-9 reductions; no RECIST response; intrapleural protocol under evaluation	[105,130]

RCT, randomized controlled trial; PK, pharmacokinetics; OA, osteoarthritis; KL, Kellgren–Lawrence grade; NLR, neutrophil-to-lymphocyte ratio; IL, interleukin; CRP, C-reactive protein; GI, gastrointestinal; AUC, area under the curve; Cmax, maximum plasma concentration; Tmax, time to reach Cmax; sCD40L, soluble CD40 ligand; TNF-α, tumor necrosis factor alpha; WOMAC, Western Ontario and McMaster Universities Osteoarthritis Index; VAS, visual analogue scale; PCSK9, proprotein convertase subtilisin/kexin type 9; NSAID, nonsteroidal anti-inflammatory drug; ESR, erythrocyte sedimentation rate; IV, intravenous; PSA, prostate-specific antigen; CEA, carcinoembryonic antigen; CA19-9, carbohydrate antigen 19-9; RECIST, Response Evaluation Criteria in Solid Tumors; miRNA, microRNA.

**Table 3:** Emerging nanotechnological and experimental systems on Echinacea formulations.

Formulation/Technology	Type of System	Experimental Model/Application	Main Outcomes (Summary)	Key Reference
Echinacea extract–chitosan–silica nanoparticles	Polymer–inorganic hybrid nanoparticles (~100–200 nm)	In vitro characterization; simulated GI release; protective effect models	Improved stability of phenolics; sustained release; favorable particle size and zeta potential	[150]
Chitosan–silica *E. purpurea* nanoparticles (CSE)	Polymer–inorganic hybrid nanoparticles (~100–150 nm)	In vivo (male rats exposed to phthalates; reproductive/oxidative stress model)	Restored antioxidant enzyme levels (SOD, CAT, GPx); reduced lipid peroxidation and testicular injury; improved sperm parameters and histology; no observed toxicity	[142]
Liposomes loaded with *E. purpurea* extract	Phosphatidylcholine liposomes (~180 nm)	In vitro antioxidant and release studies	High encapsulation efficiency (~78%); controlled release of phenolics (24 h); improved antioxidant stability; potential topical/immunoprotective application	[132]
Phytosynthesized silver nanoparticles using *E. purpurea* extracts	Green-synthesized metallic nanoparticles (AgNPs, 15–30 nm)	In vitro antimicrobial and antioxidant assays	Spherical AgNPs stabilized by plant metabolites; strong antibacterial activity (*S. aureus*, *E. coli*); high antioxidant capacity (DPPH, ABTS); non-toxic to normal cells	[150]
Green-synthesized silver nanoparticles from *E. purpurea*	Biogenic metallic nanoparticles (AgNPs, ~20–40 nm)	In vitro antioxidant and characterization study	Spherical AgNPs confirmed by UV–Vis, FTIR, SEM and XRD; strong DPPH radical scavenging and reducing power; green synthesis using ethanolic *E. purpurea* extract; stable dispersion and low cytotoxicity potential	[143]
*E. purpurea-mediated hematite nanoparticles (α-HNPs)*	Green-synthesized iron oxide nanoparticles (Fe_2_O_3_, ~25–35 nm)	In vitro physicochemical and biocompatibility assays	Successful bio-reduction and stabilization of Fe^3+^ to α-Fe_2_O_3_ using *E. purpurea* extract; crystalline spherical particles confirmed by XRD, TEM, FTIR; high antioxidant and catalytic activities; excellent hemocompatibility and cytocompatibility in fibroblast cultures	[145]
Electrosprayed nanoparticles containing *E. purpurea* hydroalcoholic extract	Polymeric nanoparticles prepared by electrospraying (~100–200 nm)	In vivo *study* in male Wistar rats (immune stimulation model)	Increased levels of pro-inflammatory cytokines (IL-1β, IL-6, TNF-α) and immunoglobulins (IgG, IgM); mild splenic hyperplasia indicating immune activation; safe dose range without organ toxicity	[146]
Electrospun keratin mats with *E. purpurea* extract and biosynthesized silver nanoparticles	Keratin-based polymeric nanofibers (electrospun, ~200–400 nm) incorporating Echinacea extract and AgNPs	In vitro physicochemical, antimicrobial, and cytocompatibility assays	Successfully fabricated uniform keratin nanofibers embedding *E. purpurea* extract and AgNPs; confirmed stability (SEM, FTIR, XRD); strong antibacterial activity against S. aureus and E. coli; maintained fibroblast viability (> 90%); potential for wound-healing and dermal delivery applications	[147]

**Table 4:** Comparative summary of pharmacological and technological characteristics of *C. longa* and *E. purpurea*.

Aspect	*C. longa*	*E. purpurea*	References
**Main bioactives**	Curcuminoids (curcumin, demethoxycurcumin, bisdemethoxycurcumin)	Alkylamides, caffeic acid derivatives, polysaccharides	[3,18]
**Immune targets**	NF-κB, MAPK, JAK/STAT, Nrf2, COX-2	TLR2/4, CB2, COX/LOX, cytokine modulation	[5,10,19]
**Regulation of COX/LOX enzymes**	Inhibits COX-2 expression and iNOS; reduces prostaglandin synthesis	Partial inhibition of COX and LOX; decreases prostaglandins and leukotrienes	[3,18,46]
**Predominant effects**	↓ IL-6, ↓ TNF-α, ↑ Treg, M1→M2 macrophage polarization	↑ NK activity, enhanced innate immunity, antiviral response	[18,19]
**Clinical focus**	Chronic inflammation, metabolic and degenerative disorders	Acute respiratory tract infections, immune support	[5,46]
**Limitations**	Poor solubility, low bioavailability	Variability in extract composition	[3,10]
**Technological advances**	Phytosomes, nanoemulsions, piperine co-formulation	Nanoencapsulation, polymeric matrices, green extraction	[5,10]
**Safety**	Excellent; minor gastrointestinal discomfort	Excellent; rare allergic reactions	[3]

## Data Availability

No new data were created or analyzed in this study. Data sharing is not applicable to this article.
